# Enhancing Micronutrient Availability Through Humic Substances and Vermicompost While Growing Artichoke Plants in Calcareous Soil: Insights from a Two-Year Field Study

**DOI:** 10.3390/plants14081224

**Published:** 2025-04-16

**Authors:** Mohamed Hafez, Zhao Zhang, Mahmoud Younis, Mahmoud A. Abdelhamid, Mohamed Rashad

**Affiliations:** 1 Land and Water Technologies Department, Arid Lands Cultivation Research Institute (ALCRI), City of Scientific Research and Technological Applications (SRTA-City), New Borg El-Arab 21934, Egypt; marashad2@gmail.com; 2 Key Laboratory of Agricultural Information Acquisition Technology, Ministry of Agriculture and Rural Affairs, China Agricultural University, Beijing 100083, China; zhaozhangcau@cau.edu.cn; 3 Chair of Dates Industry and Technology, Department of Agricultural Engineering, College of Food and Agricultural Sciences, King Saud University, P.O. Box 2460, Riyadh 11451, Saudi Arabia; myounes@ksu.edu.sa; 4 Agricultural Engineering Department, Faculty of Agriculture, Ain Shams University, Cairo 11241, Egypt; mahmoudabdelhamid@agr.asu.edu.eg

**Keywords:** vermicompost, iron chlorosis, humic substances, artichoke, recycling, calcareous

## Abstract

Calcareous soil poses challenges for crop production due to the limited availability of micronutrients in insoluble forms. This study evaluated various organic and biological treatments for managing deficiencies in iron, zinc, and manganese in artichoke (*Cynara scolymus* L.) grown in calcareous soil over two seasons (2023 and 2024). A randomized complete block design (RCBD) was employed, with 24 plots (5 × 8 m^2^ each) receiving the following five treatments: mineral fertilizer, humic substances, ALCRI-anti chlorosis, ALCRI-vermicompost, and ALCRI-bio-help. Each treatment was replicated three times. In the 2023 season, significant increases in micronutrient levels were observed following the application of the organic and biological treatments, particularly ALCRI-vermicompost and humic substances. Compared to the control group, the iron content (Fe^2+^) increased by 57.1%, reaching 715.6%. Zinc (Zn^2+^) rose by 66.1% to 686.4%, while manganese (Mn^2+^) and copper (Cu^2+^) increased by 56.9% to 685.2% and 44.9% to 673.4%, respectively. These positive trends continued into the 2024 season, with Fe^2+^ showing even greater gains of 103.4%, peaking at 824.0% in the plots treated with the ALCRI-vermicompost and humic substances. Zn^2+^ and Mn^2+^ displayed more modest increases of 36.9% and 58.0%, while Cu^2+^ exhibited a remarkable rise of 50.7%, reaching 861.2%, particularly for the ALCRI-anti chlorosis treatments. The results indicate that the application of vermicompost fertilizer, alone or in combination with humic substances, significantly enhanced the soil structure, as confirmed by the SEM examination, which revealed increased porosity and improved aggregation. These consistent improvements over two seasons strongly support the effectiveness of organic and biological treatments in enriching soil with essential micronutrients.

## 1. Introduction

Calcareous soils, which are commonly found in arid and semi-arid regions, are characterized by elevated pH levels and high calcium carbonate contents [1,2]. These conditions pose significant challenges for agricultural productivity, particularly regarding the availability of essential micronutrients [3,4,5,6,7]. Micronutrients such as iron (Fe), zinc (Zn), manganese (Mn), and copper (Cu) play critical roles in plant growth, influencing physiological processes, such as photosynthesis, respiration, and nutrient uptake [6,7,8]. However, in calcareous soils, these micronutrients often exist in insoluble forms, rendering them unavailable for plant uptake and leading to deficiencies that can severely impact crop health and yield [9,10,11,12,13,14].

Artichoke (*Cynara scolymus* L.), a highly valued perennial vegetable, is increasingly recognized for its nutritional benefits and potential health-promoting properties [15]. However, it is particularly vulnerable to micronutrient deficiencies, especially in soils with high pH levels and low organic matter content [16,17]. Deficiencies in essential micronutrients, such as Fe, Zn, and Mg, can adversely affect physiological functions, leading to stunted growth, reduced yield, and compromised quality of the edible parts, which are critical for consumer acceptance and marketability [6,18]. Consequently, addressing these micronutrient deficiencies is vital for optimizing artichoke production and ensuring the sustainability of cultivation practices in regions where this crop is a dietary staple [7].

Hafez et al. [3] found that the application of organic and biological amendments can improve the availability of micronutrients in calcareous soils. These amendments can alter soil chemistry and structure, enhancing nutrient solubility and facilitating plant uptake. Understanding the interactions between soil properties and nutrient availability is crucial for developing effective management strategies that promote micronutrient enrichment and improve artichoke productivity [7,8,18,19].

Spent grain is an organic waste that contains high contents of nitrogen, phosphorous, potassium, and other essential micronutrients [20,21,22,23]. In addition, it also contains a high level of carbohydrates, which makes it difficult for most microorganisms to break down. For the production of vermicompost fertilizers, red wigglers, which are able to efficiently break down spent grain, have been used producing a high-quality vermicompost [20,21].

Spent grain represents a significant opportunity for sustainable resource utilization, and it is crucial to acknowledge that its improper management can lead to substantial environmental and human health concerns [20]. However, the improper use and disposal of organic wastes can result in various negative impacts, including pollution, greenhouse gas emissions, water contamination, and proliferation of diseases vectors.

The reintroduction of vermicompost and humic substances into soil improves its physicochemical properties which, in turn, enhances the soil microbiome diversity and intensity, increases oxygen availability, improves water retention capacity, and reduces soil compaction [1,16]. By adopting this approach in calcareous soil, we can move toward a more sustainable agricultural system that promotes environmental preservation, reduces pollution, and ensures long-term food security. Humic substances, a group of naturally occurring organic compounds that are found in soil, have been extensively studied for their potential to enhance the nutrients richness in the soil and plant productivity [3,4,5].

In addition, the application of humic substances to the soil induces the nutrient uptake, permeability of the root cell wall, activate the plant growth mechanisms and root architecture [24]. Humic substances represent around 60% of the soil organic matter and are responsible for various soil chemical reactions; however, they are relatively difficult to be degraded by soil microbiome due their extreme structural heterogeneity [25].

Diazotrophs are nitrogen-fixing organisms that have the ability to convert atmospheric nitrogen into ammonium, which is an available form to be utilized by the plants, via nitrogenase enzyme [26]. Members of the genus *Azotobacter* represent one of the most important groups of diazotrophic bacteria [27]. In addition to their nitrogen-fixing ability, they have the ability to produce plant growth regulators, such as indole acetic acid and multiple organic acids, which contribute in their phosphate solubilization activity [27]. Moreover, they can improve the nutrient uptake by the plant and protect it against the stress factors [21,28].

Iron, zinc, and manganese are essential micronutrients that play crucial roles in plant growth as cofactors of various metabolic enzymes that regulate a plethora of physiological functions [29]. Iron deficiency can occur when plants are unable to meet their physiological requirements due to its unavailability in the soil, where it occurs in the form of poorly soluble oxides, especially in an alkaline soil [16]. Because of the disruption in chlorophyll synthesis, iron deficiency causes chlorosis of plant leaves, which is frequently observed in calcareous soils [30]. This issue highlights the need to address iron deficiency in calcareous soils through various strategies, such as the application of iron chelates or organic amendments, foliar application of iron fertilizers, and the development of crop varieties with improved iron uptake and utilization efficiency. In a recent study, the impact of spraying the humic substances and the microelements solution on iron chlorosis in tomato and artichoke plants grown on calcareous soil was investigated. The study indicated that these treatments significantly increased the Fe^2+^, Zn^2+^, and Mn^2+^ contents in the soil and artichoke, as well as improved the chlorophyll content and lowered chlorosis symptoms in artichoke plants. A hypothesis for the study was whether the application of different soil treatments (mineral fertilizer, humic substances, trace element solution, vermicompost, and humic substances supplemented with *Azotobacter* bacteria) significantly enhances the growth, yield, and micronutrient availability in artichoke plants compared to the control treatment.

This study aimed to evaluate the effectiveness of various organic, low-cost treatments (mineral fertilizer, magic plant fertilizer, ALCRI-anti chlorosis, ALCRI-vermicompost, and ALCRI-bio-help) as soil treatments and a trace elements solution (ALCRI-anti chlorosis) as a plant foliar treatment to reduce iron chlorosis and enhance the growth and yield of artichoke plants grown in calcareous soil, as well as to improve the soil content of micronutrients over two seasons.

## 2. Results

### 2.1. Iron Content of the Soil

**Figure 1:**
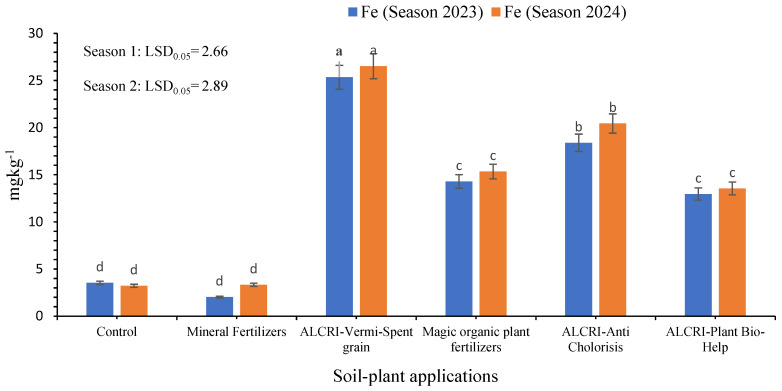
Effect of the humic substances, vermicompost, and microelement solution on the iron (Fe^2+^) content in calcareous soil over two growing seasons (2023 and 2024). In the graph, no significant difference among values with the same letter was found, according to Tukey’s HSD test (*p* ≤ 0.05). Each value represents the mean of three replicates ± SE. Control: non-treated soil; mineral fertilizer: treated with the inorganic nitrogen, phosphorus, and potassium fertilizers; magic plant fertilizer: treated with the humic substance solution at 5 L 50 kg^−1^ of seeds and soil; ALCRI-anti chlorosis: treated with the trace element solution at 4.76 L ha^−1^; ALCRI-vermi-spent grain: treated with vermicompost at 3.57-ton ha^−1^; and ALCRI-bio-help: treated with humic substances and *Azotobacter* at 5 L 50 kg^−1^ of seeds and 10 L/ha added to the soil three times with water irrigation.

Figure 1 illustrates the Fe^2+^ content in calcareous soil in response to the six applied treatments. The study showed that the content of Fe^2+^ in the soil samples significantly varied among the treatments.

The control treatment had an average content of 3.53 mg kg^−1^ in 2023, which decreased slightly to 3.22 mg kg^−1^ in 2024. This suggests that without fertilizer application, the Fe^2+^ content in the soil remained relatively low and stable over the two seasons. The plots treated with mineral fertilizers had an Fe^2+^ content of 2.021 mg kg^−1^ in 2023, which then increased to 3.33 mg kg^−1^ in 2024. This indicates that the mineral fertilizers were able to enhance the Fe^2+^ content in the soil, but the increase was not as substantial as the bio-organic fertilizer treatments.

Compared to the control, the application of the ALCRI-vermi-spent grain (vermicompost) treatment exhibited the highest increment (615.6%) in Fe^2+^ content, recording 25.340 mg kg^−1^ in 2023. In 2024, the Fe^2+^ content in the ALCRI-vermicompost-treated plots further increased to 26.522 mg kg^−1^. This demonstrates the exceptional effectiveness of the ALCRI-vermicompost bio-organic fertilizer in significantly boosting the availability of Fe^2+^ in calcareous soil. The other bio-organic fertilizer treatments also showed promising results, such as ALCRI-anti chlorosis, as follows: 18.39 mg kg^−1^ in 2023, increasing to 20.440 mg kg^−1^ in 2024; humic substances: 14.29 mg kg^−1^ in 2023, increasing to 15.34 mg kg^−1^ in 2024; ALCRI-plant bio-help: 12.95 mg kg^−1^ in 2023, increasing to 13.540 mg kg^−1^ in 2024.

The data indicate that the bio-organic fertilizer treatments, particularly ALCRI-vermicompost, were more effective in increasing the Fe^2+^ content in the calcareous soil compared to the control and mineral fertilizer treatments. The sustained increase in Fe^2+^ levels from 2023 to 2024 further highlights the sustained benefits of the bio-organic fertilizers in improving soil nutrient availability.

### 2.2. Zinc (Zn) Content of the Soil

**Figure 2:**
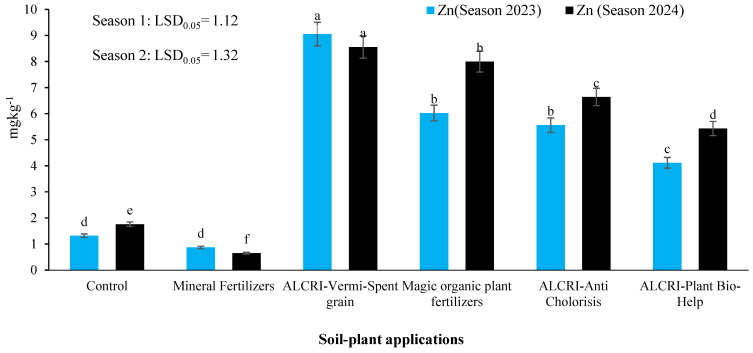
Effect of the humic substances, vermicompost, and microelement solution on zinc (Zn^2+^) contents in calcareous soil over two growing seasons (2023 and 2024). In the graph, no significant difference among values with the same letter was found, according to Tukey’s HSD test (*p* ≤ 0.05). Each value represents the mean of three replicates ± SE. Control: non-treated soil; mineral fertilizer: treated with inorganic nitrogen, phosphorus, and potassium fertilizers; magic plant fertilizer: treated with the humic substances solution at 5 L 50 kg^−1^ of seeds and soil; ALCRI-anti chlorosis: treated with the trace elements solution at 4.76 L ha^−1^; ALCRI-vermi-spent grain: treated with vermicompost at 3.57-ton ha^−1^; and ALCRI-bio-help: treated with humic substances with *Azotobacter* at 5 L 50 kg^−1^ of seeds and 10 L/ha added to the soil three times with water irrigation.

The data presented in Figure 2 illustrate the levels of Zn in the calcareous soil under the various treatment regimens over two growing seasons. In the control plots, the Zn content increased from 1.320 mg kg^−1^ in 2023 to 1.760 mg kg^−1^ in 2024, indicating that without fertilizer application, the availability of Zn in the soil was limited. The application of mineral fertilizers led to a decrease in Zn content, declining from 0.872 mg kg^−1^ in 2023 to 0.650 mg kg^−1^ in 2024, suggesting that the mineral fertilizers were not effective at maintaining or enhancing Zn’s availability in the soil.

In contrast, the bio-organic fertilizer treatments demonstrated a remarkable impact on the Zn levels. The ALCRI-vermicompost treatment exhibited the highest Zn content, decreasing slightly from 9.05 mg kg^−1^ in 2023 to 8.56 mg kg^−1^ in 2024, but remaining substantially higher compared to the control and mineral fertilizer treatments. The other bio-organic fertilizer treatments, including ALCRI-anti chlorosis, humic substances, and ALCRI-plant bio-help, also demonstrated significant enhancements in Zn content over the two-year period, with levels ranging from 4.11 mg kg^−1^ to 8.00 mg kg^−1^, clearly outperforming the control and mineral fertilizer treatments.

These findings suggest that the bio-organic fertilizer applications, particularly the ALCRI-vermicompost treatment, were highly effective at improving the availability and content of Zn in the calcareous soil over the two growing seasons. This highlights the potential of these bio-organic amendments as sustainable approaches to enhancing soil fertility and nutrient management in calcareous agricultural systems.

### 2.3. Manganese Content of the Soil

**Figure 3:**
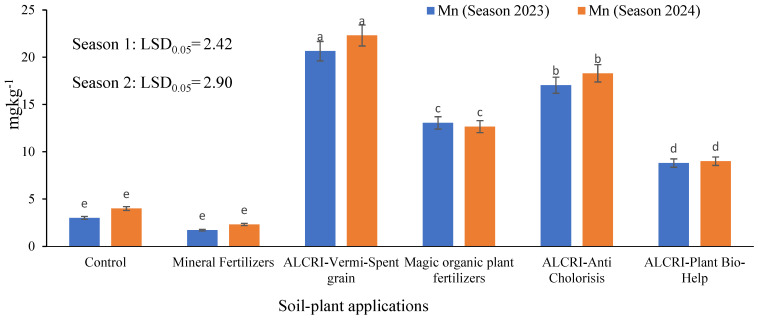
Effect of the humic substances, vermicompost, and microelements solution on the manganese (Mn^2+^) content in calcareous soil over two growing seasons (2023 and 2024). In the graph, no significant difference among values with the same letter was found, according to Tukey’s HSD test (*p* ≤ 0.05). Each value represents the mean of three replicates ± SE. Control: non-treated soil; mineral fertilizer: treated with inorganic nitrogen, phosphorus, and potassium fertilizers; magic plant fertilizer: treated with the humic substances solution at 5 L 50 kg^−1^ of seeds and soil; ALCRI-anti chlorosis: treated with the trace elements solution at 4.76 L ha^−1^; ALCRI-vermi-spent grain: treated with the vermicompost at 3.57-ton ha^−1^; and ALCRI-bio-help: treated with humic substances with *Azotobacter* at 5 L 50 kg^−1^ of seeds and 10 L/ha added to the soil three times, with water irrigation. The graphs depict the mean ± SE (standard error).

The data presented in the Figure 3 illustrate the levels of Mn in calcareous soil under the various treatment regimens over two growing seasons. In the control plots, the Mn content increased from 3.013 mg kg^−1^ in 2023 to 4.00 mg kg^−1^ in 2024, indicating a moderate increase in Mn availability over the two growing seasons. The application of mineral fertilizers led to a lower Mn content, rising from 1.714 mg kg^−1^ in 2023 to 2.32 mg kg^−1^ in 2024, suggesting that the mineral fertilizers were not effective in significantly enhancing Mn availability in the soil. In contrast, the bio-organic fertilizer treatments demonstrated a more substantial impact on the Mn levels. The ALCRI-vermicompost treatment exhibited the highest Mn content, increasing from 20.64 mg kg^−1^ in 2023 to 22.30 mg kg^−1^ in 2024, a significant improvement over the control and mineral fertilizer treatments.

The other bio-organic fertilizer treatments, including ALCRI-anti chlorosis, humic substances, and ALCRI-plant bio-help, also showed notable increases in Mn contents over the two-year period, with the levels ranging from 9.00 mg kg^−1^ to 18.30 mg kg^−1^, outperforming the control and mineral fertilizer treatments. These findings suggest that the bio-organic fertilizer applications, particularly the ALCRI-vermicompost treatment, were effective in enhancing the availability and content of Mn in the calcareous soil over the two growing seasons. This indicates the potential of these bio-organic amendments to improve soil fertility and nutrient management in calcareous agricultural systems.

### 2.4. Copper Content of the Soil

**Figure 4:**
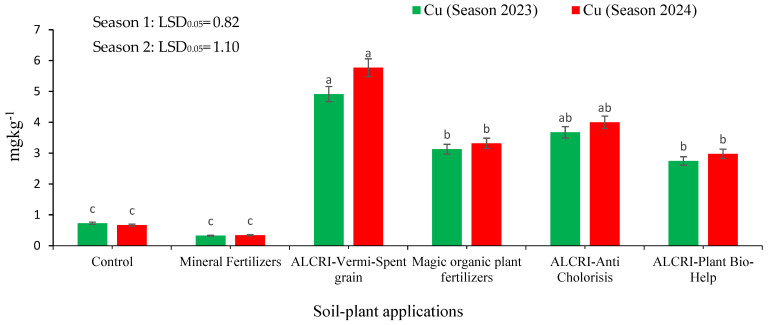
Effect of the humic substances, vermicompost, and microelements solution on the copper (Cu^2+^) contents in the calcareous soil over two growing seasons (2023 and 2024). In the graph, no significant difference among values with the same letter was found, according to Tukey’s HSD test (*p* ≤ 0.05). Each value represents the mean of three replicates ± SE. Control: non-treated soil; mineral fertilizer: treated with the inorganic nitrogen, phosphorus, and potassium fertilizers; magic plant fertilizer: treated with the humic substances solution at 5 L 50 kg^−1^ of seeds and soil; ALCRI-anti chlorosis: treated with the trace elements solution at 4.76 L ha^−1^; ALCRI-vermi-spent grain: treated with the vermicompost at 3.57-ton ha^−1^; and ALCRI-bio-help: treated with humic substances with *Azotobacter* at 5 L 50 kg^−1^ of seeds and 10 L/ha added to the soil three times with water irrigation. The graphs depict the mean ± SE (standard error).

The data presented in Figure 4 show the Cu content in calcareous soil under different treatment regimens over two growing seasons. In the control plots, the Cu content decreased from 0.73 mg kg^−1^ in 2023 to 0.67 mg kg^−1^ in 2024, indicating a slight decline in Cu availability over the two growing seasons. The application of mineral fertilizers led to a lower Cu content, increasing from 0.32 mg kg^−1^ in 2023 to 0.34 mg kg^−1^ in 2024, suggesting that the mineral fertilizers were not effective at significantly enhancing the Cu availability in the soil. In contrast, the bio-organic fertilizer treatments demonstrated a more substantial impact on the Cu levels. The ALCRI-vermicompost treatment exhibited the highest Cu content, increasing from 4.91 mg kg^−1^ in 2023 to 5.77 mg kg^−1^ in 2024, a significant improvement over the control and mineral fertilizer treatments.

The other bio-organic fertilizer treatments, including ALCRI-anti chlorosis, humic substances, and ALCRI-plant bio-help, also showed notable increases in Cu contents over the two-year period, with the levels ranging from 3.13 mg kg^−1^ to 4.00 mg kg^−1^, outperforming the control and mineral fertilizer treatments. These findings suggest that the bio-organic fertilizer applications, particularly the ALCRI-vermicompost treatment, were effective at enhancing the availability and content of Cu in the calcareous soil over the two growing seasons. This indicates the potential of these bio-organic amendments to improve soil fertility and nutrient management in calcareous agricultural systems.

The Cu^2+^ contents in the calcareous soil, in response to the six applied treatments, are illustrated in Figure 3. The contents of Cu^2+^ in the soil samples significantly varied among the treatments. The mineral treatment resulted in the lowest content of Cu^2+^ (0.73 mg kg^−1^). The highest content of Cu^2+^ was found with the ALCRI-vermicompost treatment (4.91 mg kg^−1^), followed by the ALCRI-anti chlorosis and the magic organic plant fertilizers, recording 3.68 and 3.13 mg kg^−1^, respectively. The application of ALCRI-plant bio-help enhanced the Cu^2+^ content, with 2.75 mg kg^−1^ recorded. No significant difference was observed between the mineral fertilizers and the control treatment.

### 2.5. Scanning Electron Microscopic (SEM) Observations

**Figure 5:**
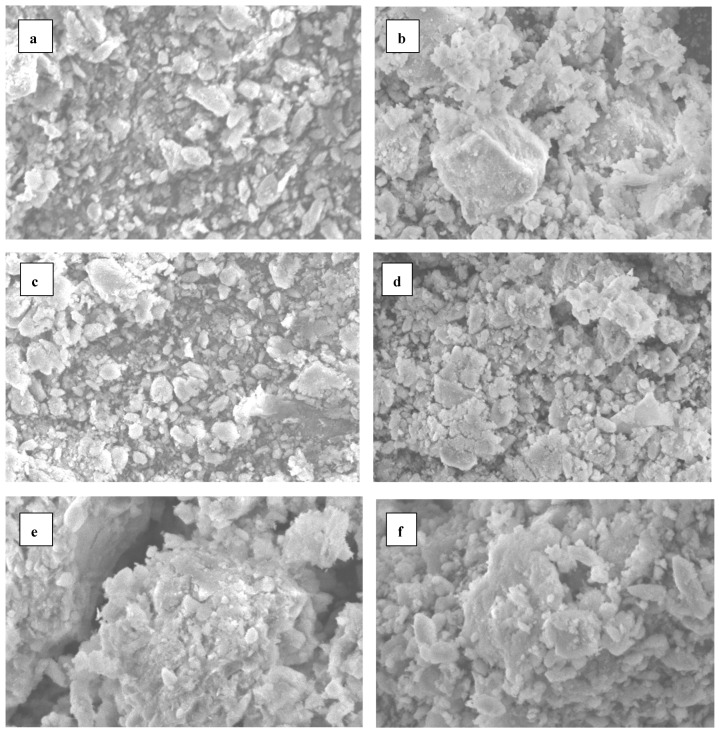
SEM micrographs showing the calcareous soil structure in response to the various applied treatments: (**a**) non-treated soil; (**b**) treated with the mineral fertilizers; (**c**) treated with the vermi-spent grain; (**d**) treated with magic organic plant fertilizers; (**e**) treated with ALCRI anti-chlorosis; (**f**) treated with ALCRI-plant bio-help. Bar = 5 µm.

The SEM observations of the soil structure, in response to the tested treatments, revealed distinct characteristics among the various treatments. For the control (i.e., non-treated soil), the SEM image displayed a compact and tightly packed soil matrix with low porosity, indicating limited air and water movement, which can restrict root growth and microbial activity (Figure 5). In the mineral fertilizer treatment, there was an observable improvement in the soil structure compared to the control. The SEM image likely showed increased porosity and better aggregation, indicating enhanced aeration and water infiltration, creating a more favorable environment for plant roots. Soil treated with the magic plant fertilizer exhibited considerable improvement in porosity and structural stability, with evidence of microbial activity benefiting overall soil health. The ALCRI-anti chlorosis treatment demonstrated similar positive effects, promoting both aggregate stability and nutrient availability.

Conversely, soil treated with ALCRI-vermicompost showed improved aggregation and microbial diversity, enhancing water retention and root growth potential. However, the excessive application of vermicompost could lead to compaction issues, as observed in treatments with higher concentrations. The ALCRI-bio-help treatment revealed enhanced microbial activity that positively affected the soil structure at lower concentrations. However, at higher application rates, signs of compaction and reduced porosity were evident, suggesting that excessive use of both bacteria and humic substances could adversely affect the soil structure, potentially negating the benefits of increased microbial activity and organic matter. Generally, these observations highlight the importance of the dosage and application methods in optimizing the soil structure and fertility through various treatments.

### 2.6. Biomass of Artichoke Yield Indicator in Calcareous Soil

**Figure 6:**
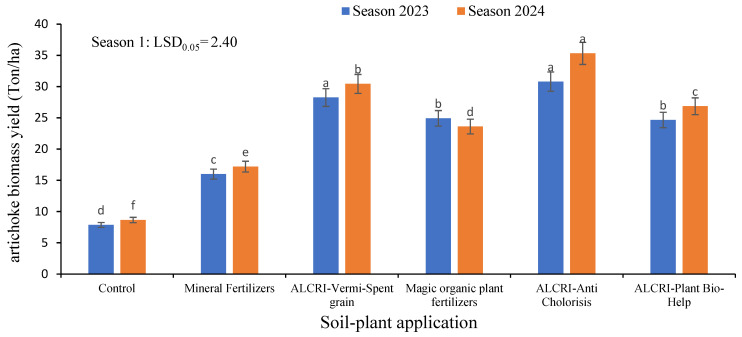
Effect of the humic substances, vermicompost, and microelements solution on the biomass yield for artichoke plant in the calcareous soil over two growing seasons (2023 and 2024). In the graph, no significant difference among values with the same letter was found, according to Tukey’s HSD test (*p* ≤ 0.05). Each value represents the mean of three replicates ± SE. Control: non-treated soil; mineral fertilizer: treated with the inorganic nitrogen, phosphorus, and potassium fertilizers; magic plant fertilizer: treated with the humic substances solution at 5 L 50 kg^−1^ of seeds and soil; ALCRI-anti chlorosis: treated with the trace elements solution at 4.76 L ha^−1^; ALCRI-vermi-spent grain: treated with vermicompost at 3.57-ton ha^−1^; and ALCRI-bio-help: treated with humic substances with *Azotobacter* at 5 L 50 kg^−1^ of seeds and 10 L/ha added to the soil three times with water irrigation. The graphs depict the mean ± SE (standard error).

The data presented in Figure 6 show the artichoke biomass yield in calcareous soil under the various treatment regimens over two growing seasons in 2023 and 2024. As shown in the table, the control treatment had an average artichoke biomass yield of 7.86 tons/ha in 2023, which increased slightly to 8.65 tons/ha in 2024. This suggests that without any fertilizer application, the artichoke biomass yield remained relatively low and stable over the two seasons.

The plots treated with mineral fertilizers had an artichoke biomass yield of 16.0 tons/ha in 2023, which then increased to 17.20 tons/ha in 2024. This indicates that the mineral fertilizers were able to enhance the artichoke biomass yield in the soil, but the increase was not as substantial as the bio-organic fertilizer treatments.

Compared to the control, the application of the ALCRI-vermicompost bio-organic fertilizer treatment exhibited a significant increase in the artichoke biomass yield, recording 28.25 tons/ha in 2023. In 2024, the artichoke biomass yield in the ALCRI-vermicompost-treated plots further increased to 30.43 tons/ha. This demonstrates the exceptional effectiveness of the ALCRI-vermicompost bio-organic fertilizer at significantly boosting the artichoke biomass production in the calcareous soil.

The other bio-organic fertilizer treatments also showed promising results in increasing the artichoke biomass yield. The ALCRI-anti chlorosis treatment increased from 30.80 tons/ha in 2023 to 35.316 tons/ha in 2024, the Magic organic plant fertilizers treatment increased from 24.92 tons/ha in 2023 to 23.61 tons/ha in 2024, and the ALCRI-plant bio-help treatment increased from 24.65 tons/ha in 2023 to 26.85 tons/ha in 2024. The data indicate that the bio-organic fertilizer treatments, particularly the ALCRI-vermicompost and ALCRI-anti chlorosis, were more effective in increasing the artichoke biomass yield in the calcareous soil compared to the control and mineral fertilizer treatments. The sustained increase in artichoke biomass yield from 2023 to 2024 further highlights the sustained benefits of the bio-organic fertilizers in improving crop productivity in calcareous soil conditions.

### 2.7. Effect of the Micronutrients Content on Artichoke Fruit

**Figure 7:**
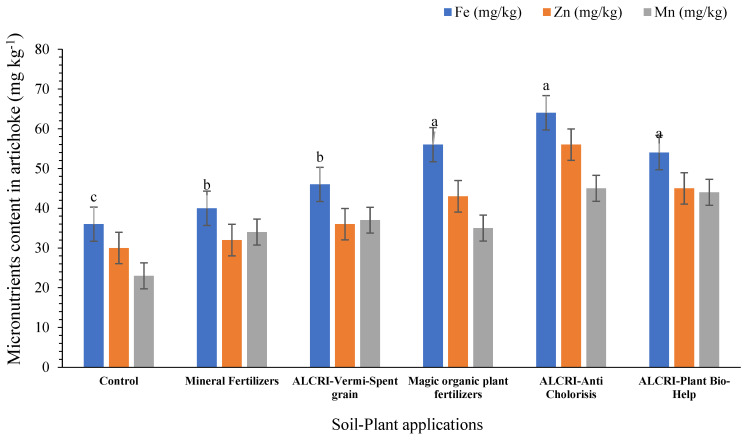
Effect of humic substances, vermicompost, and microelements solution on micronutrient content in artichoke fruit in calcareous soil for the final season of 2024. In the graph, no significant difference among values with the same letter was found, according to Tukey’s HSD test (*p* ≤ 0.05). Each value represents the mean of three replicates ± SE. Control: non-treated soil; mineral fertilizer: treated with inorganic nitrogen, phosphorus, and potassium fertilizers; magic plant fertilizer: treated with the humic substances solution at 5 L 50 kg^−1^ of seeds and soil; ALCRI-anti chlorosis: treated with the trace elements solution at 4.76 L ha^−1^; ALCRI-vermi-spent grain: treated with vermicompost at 3.57-ton ha^−1^; and ALCRI-bio-help: treated with humic substances with *Azotobacter* at 5 L 50 kg^−1^ of seeds and 10 L/ha added to the soil three times with water irrigation. The graphs depict the mean ± SE (standard error).

Figure 7 provides information on the concentrations of three micronutrients (Fe^2+^, Zn^2+^, and Mn^2+^) in artichoke fruit in response to the applied treatments after two seasons of seed sowing. Regarding Fe^2+^, Zn^2+^, and Mn^2+^, the control treatment had the lowest micronutrients content in the artichoke fruit, recording 36, 30, and 23 mg kg^−1^, respectively. The application of the mineral fertilizers slightly increased the micronutrients content in artichoke fruit compared to the control. The concentrations of Fe^2+^, Zn^2+^, Mn^2+^ in the artichoke fruit under the mineral fertilizer treatment were 40, 32, and 34 mg kg^−1^, respectively.

However, the treatments ALCRI-vermicompost, magic organic plant fertilizers, ALCRI-anti chlorosis, and ALCRI-plant bio-help showed significantly higher micronutrient contents in artichoke fruit compared to the control and the mineral fertilizer treatments. Under the ALCRI-vermicompost treatment, the concentrations of Fe^2+^, Zn^2+^, Mn^2+^ in artichoke fruit were measured as 46, 36, and 37 mg kg^−1^, respectively.

With the magic organic plant fertilizers treatment, artichoke fruit exhibited even higher micronutrient contents. The concentrations of Fe^2+^, Zn^2+^, and Mn^2+^ in artichoke fruit were recorded as 56, 43, and 35 mg kg^−1^, respectively. Similarly, the ALCRI-anti chlorosis treatment resulted in increased contents of Fe^2+^, Zn^2+^, and Mn^2+^ in artichoke fruit, recording 64, 56, and 45 mg kg^−1^, respectively. The ALCRI-plant bio-help treatment showed higher contents of Fe^2+^, Zn^2+^, and Mn^2+^ in the artichoke fruit, compared to the control treatment, recording 54, 45, and 44 mg kg^−1^, respectively.

## 3. Discussion

The use of biofertilizers and vermicompost is an eco-friendly alternative to mineral fertilizers in agriculture [7,16,31]. These organic products have been shown to be effective at enhancing plant production and soil health, as well as mitigating the negative environmental effects due to chemical fertilizers. However, to achieve the best results, the appropriate biofertilizer or vermicompost should be selected based on the types of crop and soil. The results obtained in this study indicate that adding ALCRI-vermicompost to the calcareous soil increased its micronutrient contents and promoted artichoke growth and production more than the NPK fertilizers over the 2023 and 2024 seasons. The ALCRI-anti chlorosis treatment also had a positive effect on the Fe^2+^ content in the calcareous soil, especially in the 2023 season compared with the 2024 season. In addition, the use of the diazotrophic bacteria *Azotobacter* sp. as a plant growth-promoting treatment increased the artichoke production in the 2024 season.

The application of the nitrogen-fixing bacteria played an enhancing role in providing a nitrogen source for the plant, as well as improving the soil properties, over the two seasons [17]. In contrast, the effects of the application of the mineral fertilizers on the micronutrient contents of the calcareous soil and on artichoke growth were found to be weak and not suitable for artichoke cultivation in calcareous soils over two seasons. In this regard, it was found that the addition of the ALCRI-vermicompost and magic organic plant fertilizers significantly enhanced the soil’s Fe content and alleviated the iron chlorosis in the artichoke plants compared with the control and mineral fertilizer treatments in the 2024 season. These findings are consistent with previous studies suggesting that compost can enhance iron’s availability in calcareous soils by improving their biological functioning, aeration, and availability [6,8,12,18,19,31].

The vermicompost application improved the soil’s health by enhancing the microbial activity and increasing the organic matter content, leading to better nutrient availability in plants during the 2023 season compared to the 2024 season. This led to an increment in the Fe^2+^ content in the soil and, subsequently, in the plant. Adding magic organic plant fertilizers, with their organic composition and nutrient contents, facilitated improved nutrient uptake and utilization by the plants. Hafez et al. [4,5] found that the soil organic matter has a crucial role in enhancing the iron availability, as well as mitigating the risks of iron chlorosis. Iron can be chelated by organic acids, humic molecules, and siderophores, making it more accessible to plants [31]. The presence of a diverse microflora, including siderophore-producing microbes, has been shown to be crucial for optimal iron nutrition in various plants [9,10,11,12,13]. Furthermore, enhancing aeration and organic matter content in alkaline soils can reduce the transformation of ferrihydrite, the precursor for the majority of iron oxides in soil, into more complicated iron oxides [3].

In this study, the application of ALCRI-vermicompost, magic organic plant fertilizers, and ALCRI-anti chlorosis was found to significantly improve the Zn^2+^ content in the soil compared to the control and mineral fertilizers, with the second season (i.e., 2024) better than the first season (i.e., 2023). Vermicompost and organic fertilizers contain high levels of micronutrients, including Zn^2+^, which can improve the availability of this nutrient in the soil. Additionally, the soil organic matter plays a crucial role in enhancing the soil structure and water-holding capacity, resulting in the enhancement of the nutrient uptake by the plants [3,20,32]. At the same time, the application of ALCRI-vermicompost, magic organic plant fertilizers, and ALCRI-anti chlorosis significantly improved the soil’s contents of Mn^2+^ and Cu^2+^ in the two seasons of 2023 and 2024 compared to the control and mineral fertilizers. Vermicompost and organic fertilizers contain high levels of Mn^2+^ and Cu^2+^, which can improve its availability in the soil [21,23].

Furthermore, the application of the vermicompost and humic substances can improve the soil’s pH, which can help in the release of Mn^2+^ from soil minerals and make it more available to plants [33]. Many previous studies have provided evidence supporting the enhancing effects of the organic amendments on the soil properties and plant micronutrient management [34]. Hale et al. [21] reported that soils amended with organic and humic substances exhibited a significant enhancement in their physicochemical and biological properties. Carricondo-Martínez et al. [34] reported the successful integration of different crop residues into the soil through vermicompost and plant growth-promoting rhizobacteria, which resulted in a tomato yield with high nutritional quality. These amendments were found to increase the microbial biomass by 127% under full irrigation and 157% under deficit-irrigation conditions compared to the control. Li et al. [29] investigated the effects of organic amendments and vermicompost on the soil microbiome and found that they enhanced bacterial community interactions. The authors also found that vermicompost’s addition improved the activity of the antioxidant enzymes polyphenol oxidase and catalase in plants, mitigating the negative impacts of the soil’s salinity. Ali et al. [31] reported significant improvements in soil properties in response to the application of vermicompost. The increase in the micronutrient availability in plants with the addition of organic and bio-organic amendments, as observed in the present study, can be attributed to the cycling of micronutrients in the soil [35,36]. Additionally, these amendments may have stimulated the activity of specific Fe transporters in the plant’s roots, resulting in enhancements in the micronutrient uptake. Overall, these studies supported the use of organic amendments in improving soil properties, nutrient availability, and plant growth, providing sustainable solutions for agricultural practices.

The ALCRI-anti chlorosis foliar application treatment, which was specifically designed to address iron chlorosis, showed the highest increase in micronutrient concentrations, indicating its effectiveness in the management of Fe^2+^, Zn^2+^, and Mn^2+^ deficiencies over two seasons in 2023 and 2024. Iron chlorosis occurs when plants are unable to take up an adequate amount of iron, leading to Fe^2+^ deficiency [9,10,11,13].

By applying the ALCRI-anti chlorosis treatment, the availability of iron, zinc, and manganese improved, resulting in higher micronutrient concentrations in the artichoke fruit, especially in the 2024 season. This treatment contains specific formulations and compounds that enhance the uptake and utilization of these micronutrients by the plants providing healthier growth and productivity. These results are in agreement with those of [36,37]. The ALCRI-plant bio-help treatment, which likely involved the application of plant growth-promoting substances, improved the nutrient assimilation and uptake by the plants, leading to higher micronutrient concentrations in the fruit. Overall, these findings highlight the efficacy of the various treatments at increasing the micronutrient contents in the artichoke fruits, offering potential solutions to address micronutrient deficiencies and improve the nutritional quality of harvested crops over two seasons [6,8,18,19].

The scanning electron microscopy (SEM) analysis of the soil samples revealed significant differences in the soil structures based on the various treatments applied. The untreated control soil displayed a compact, low-porosity structure, which limited the root penetration and hindered water movement. This suggests minimal microbial activity and insufficient porosity, both of which are critical for a healthy soil ecosystem [37].

In contrast, the application of mineral fertilizer significantly improved the soil structure. The SEM images indicate enhanced particle aggregation and increased porosity, creating a more open structure that facilitated better water infiltration and promoted root growth [38]. The application of the magic plant fertilizer, consisting of a humic substances solution, also demonstrated positive effects on the soil structure. The SEM observations revealed an improved soil aggregation and microbial activity, contributing to increased porosity and enhancing the overall health of the soil.

The three applications of the ALCRI-anti chlorosis treatment during plant growth further enhanced the soil structure. The SEM analysis indicated better particle interaction, leading to improved aggregation and nutrient availability, which is beneficial for root development. The ALCRI-vermicompost treatment, showed a marked improvement in soil structure and microbial diversity. The SEM images reveal enhanced porosity and stability, promoting water retention and supporting root growth. The combination of ALCRI-bio-help, added to the soil three times with irrigation, produced complex results. This treatment improved the soil aggregation and microbial activity, but at higher concentrations, there were indications of reduced porosity and potential compaction, suggesting that careful management of this application is necessary to avoid adverse effects.

## 4. Materials and Methods

### 4.1. Study Site Information

The study was conducted at a research farm located in Bangr Elsokar, Alamrea city, Egypt. The experimental area covered 960 m^2^ of calcareous soil, which is classified as “Calcisols”, according to the World Reference Base for Soil Resources (WRB) 2015. It was divided into 18 plots, each measuring 5 × 8 m^2^. The experiments spanned two growing seasons, 2023 and 2024, focusing on various treatments for artichoke (*Cynara scolymus* L.) cultivation. The site is characterized by its semi-arid climate, making it suitable for artichoke production. Regular drip irrigation, totaling approximately 2600 m^3^ of water per growing season, was implemented to support crop growth throughout the study period. The soil analysis revealed a pH of 8.34, EC of 0.67 dS/m, and TN of 0.8 g kg^−1^, while the available phosphorus and potassium were found to be 7.0 mgkg^−1^ and 176.0 mgkg^−1^, respectively, indicating relatively low nutrient levels, which may require amendments for the optimal crop performance. The organic matter (OM) content was measured at 2.2 g kg^−1^. Calcium (Ca^2+^) was measured at 20 mgkg^−1^, alongside magnesium (Mg^2+^) at 12 mgkg^−1^, both essential macronutrients for plant development. The micronutrient analysis showed the level of iron (Fe^2+^) was 1.77 mgkg^−1^, zinc (Zn^2+^) was 1.12 mgkg^−1^, and manganese (Mn^2+^) was 0.71 mgkg^−1^.

### 4.2. Experimental Layout

Six treatments were applied in this experiment, namely, control (i.e., untreated), mineral fertilizer, humic substances, trace elements solution, vermicompost, and humic substances supplemented with *Azotobacter* bacteria. The type and rate of application for each treatment are presented in Table 1. For each treatment, three replicated plots were established, ensuring that each treatment was represented equally across the experimental area. All plots were arranged in a randomized complete block design to minimize potential biases and ensure that environmental variations were evenly distributed. This layout allows for a more rigorous comparison of treatment effects. A total amount of 4284 artichoke (*Cynara scolymus* L.) plants ha^−1^ was planted in the soil, with a density of 1 plant/40 cm^2^. The soil was organized into rows spaced 170 cm apart. Within each row, plants were positioned at 40 cm intervals, ensuring optimal spacing for growth. The artichoke plants were regular drip irrigated.

### 4.3. Preparation of Vermicompost (ALCRI-Vermicompost)

Vermicompost was prepared using a brewery’s spent grain organic wastes and red wigglers earthworms. The spent grain was mixed with cow dung (3:1), moistened to a water content of 60–70%, and placed in vermiculture beds with a stocking density of 10 worms/kg dry matter. The beds were kept in a shaded area and regularly moistened over two months. The resulting vermicompost was harvested, sieved, and air-dried for one week to produce a nutrient-rich organic fertilizer with a pH of 7.5. The preparation methods for the treatment analyses are presented in Table 2, Table 3 and Table 4.

### 4.4. Preparation of the Humic Substances (Magic Plant Fertilizer)

The humic substances solution was prepared by extracting the vermicompost by a 0.1 M KOH solution at 1:10 (*w*/*v*). The mixture was stirred for 24 h and centrifuged at 5000 rpm for 10 min. The supernatant was collected and the pH adjusted to 7.0; then, it was filtered through a 0.45 μm filter and stored at 4 °C for further use [22,23]. Treatment analyses preparation methods are presented in Table 2 and Table 3.

### 4.5. Preparation of the Microelements Solution (ALCRI-Anti Chlorosis)

The trace elements solution was prepared by dissolving a mixture of micronutrients in water. The mixture contained the following in 1 L of distilled water: FeSO_4_·7H_2_O (8 g L^−1^), MnSO_4_·H_2_O (2.25 g L^−1^), ZnSO_4_·7H_2_O (2 g L^−1^), CuSO_4_·5H_2_O (1.0 g L^−1^), and (NH_4_)_6_Mo_7_O_24_·4H_2_O (0.23 g L^−1^). The solution was stirred thoroughly using a magnetic stirrer until all salts were dissolved and the pH was adjusted to 6.0; then, the final volume was brought up to 5 L with distilled water. The solution was stored in a labeled, air-tight container until used [3]. The treatment analyses preparation methods are presented in Table 2 and Table 3.

### 4.6. Soil Analyses

For each treatment, soil samples were collected during planting for the chemical analysis. All soil samples were sieved to separate any roots or litter materials and air-dried at room temperature for 3 months. Three replicates were utilized for each plot. The micronutrients—iron (Fe^2+^), zinc (Zn^2+^), manganese (Mn^2+^), and copper (Cu^2+^)—were extracted using DTPA solution according to [39,40] and quantified using inductively coupled plasma (ICP) atomic emission spectroscopy [41,42]

### 4.7. Plant Analyses

The artichoke plants were collected and the biological yield was determined. The micronutrients contents (Fe^2+^, Zn^2+^, and Mn^2+^) were measured in artichoke plants as described [43,44]. Three replicates were utilized for each plot.

### 4.8. SEM Examination

Soil samples were air-dried and mounted on aluminum stubs using double-sided carbon tape after coating with gold. The samples were then examined using an SEM (JEOL-JSM-6360-LA) to visualize the soil’s microstructure at high resolution.

### 4.9. Statistical Analysis

The data obtained were statistically analyzed using SPSS software V.30 First, all data were subjected to a one-way analysis of variance. The means of the data were compared using Tukey’s HSD test (*p* ≤ 0.05).

## 5. Conclusions

The study evaluated the impacts of various organic and biological amendments on iron deficiency in artichoke plants grown in calcareous soils during the 2023 and 2024 seasons. The findings indicate that incorporating microelements through liquid fertilizers significantly influenced the micronutrient content in plants compared to mineral fertilizers, which lacked these essential elements. In the 2023 season, the applications of the ALCRI-vermicompost, ALCRI-bio-Help, and magic organic plant fertilizers and ALCRI-anti chlorosis notably improved the soil contents of iron, zinc, manganese, and copper. Furthermore, the ALCRI-anti chlorosis treatment, applied foliar, consistently increased micronutrient levels in artichoke fruits over the 2024 season. The results also demonstrate that using vermicompost fertilizer, either alone or in combination with magic plant fertilizers, significantly enhanced the soil structure. Scanning electron microscopy (SEM) analysis confirmed an increased porosity and improved aggregation, contributing to better soil health. Based on these findings, it is recommended to utilize the ALCRI-anti chlorosis solution as a foliar treatment to effectively address iron chlorosis deficiency and enhance micronutrient levels in both soil and fruit for artichoke cultivation in arid regions. However, further studies are essential to investigate the long-term effects of these products on crop yield and soil health over extended periods.

## Figures and Tables

**Table 1:** Treatments, application times, and concentrations for the two seasons.

Treatment	Description	Application Dose, Time and Concentration
Control	Untreated soil and plant	
Mineral fertilizer	Soil amendments	* 300 kg ha^−1^ of N, P, and K (20:20:20) was applied once as a soil treatment before planting
Magic plant fertilizer (humic substances solution)	Seed treatments and soil	5 L 50 kg^−1^ of seeds was added twice with seed sowing in the greenhouse and plant growth in the soil
ALCRI-anti chlorosis (trace elements solution)	Foliar and soil applications	4.76 L ha^−1^ was added three times during the plants’ growth
ALCRI-vermi-spent grain (vermicompost)	Applied as a soil treatment	3.57-ton ha^−1^ was added once during the seed sowing
ALCRI-bio-help (humic substances with *Azotobacter* sp.)	Seeds and soil treatments	5 L 50 kg^−1^ of seeds and 10 L/ha added to the soil three times with water irrigation

The selected dose of 3.57 tons ha^−1^ of vermicompost was based on the total number of plants planted per hectare, which was approximately 4284 plants. Assuming that each plant requires about 833 g of vermicompost, the total amount needed for one hectare is approximately 3570 kg. This calculation ensured that each plant received an adequate supply of organic matter to enhance the soil fertility and promote healthy growth. * The mineral fertilization was applied at a recommended dose of 300 kg/ha, as advised by the Egyptian Ministry of Agriculture (2012). This application rate was designed to provide essential nutrients for optimal plant growth and development. The specific mineral fertilizers used were a multi-component NPK formulation, which supplies nitrogen (N), phosphorus (P), and potassium (K) in balanced proportions.

**Table 2:** Chemical analysis of the soil treatments.

Treatments	pH	EC	TN	OM	TOC	Ca	Mg	Fe	Zn	Mn
		mS cm^−1^	g kg^−1^					mg kg^−1^		
ALCRI-Vermicompost	7.5	1.2	25	350	200	20	5	100	50	40
Magic Plant Fertilizer	7.0	0.8	10	300	150	15	3	80	30	20
ALCRI-Anti Chlorosis	6.0	0.5	1.0	0.00	0.00	1.0	0.5	160	40	30
ALCRI-Bio-Help	6.5	0.6	15	320	180	18	4	90	35	25

TN: total nitrogen; OM: organic matter; TOC: total organic carbon.

**Table 3:** Quantities of macro- and microelements added with the fertilizer treatments (kg/ha/yr).

Treatment	N	P	K	Fe	Zn	Mn
	N	(P_2_O_5_)	(K_2_O)			
ALCRI-Vermicompost	0.53	0.32	0.62	0.13	0.05	0.04
Magic Plant Fertilizer	0.59	0.56	0.52	0.08	0.03	0.02
ALCRI-Anti Chlorosis	0.0	0.0	0.0	0.56	0.45	0.39
ALCRI-Bio-Help	0.25	0.21	0.41	0.09	0.035	0.025
Mineral Fertilizer	60	60	60	0.0	0.0	0.0

**Table 4:** Preparation methods and properties of the soil treatments for the micronutrient availability.

Treatment	Preparation Method	Ingredients/Components	Final Properties
ALCRI-Vermicompost	Mixed brewery spent grain with cow dung in a 3:1 ratio, Moistened to a 60–70% water content, Placed in vermiculture beds with 10 worms/kg dry matter, Kept in a shaded area and regularly moistened for two months, Harvested, sieved, and air-dried for one week.	Brewery spent grain—Cow dung—red wigglers earthworms	Nutrient-rich organic fertilizer—pH: 7.5
Magic Plant Fertilizer (Humic Substances)	Extracted vermicompost using 0.1 M KOH solution in a 1:10 (*w*/*v*) ratio, Stirred for 24 h, Centrifuged at 5000 rpm for 10 min, Supernatant was collected and the pH adjusted to 7.0, and then it was filtered through 0.45 μm filter, Stored at 4 °C.	Vermicompost—0.1 M KOH solution	Humic substances solution—pH: 7.0
ALCRI-Anti Chlorosis (Microelements Solution)	Micronutrient mixture was dissolved in distilled water, It was stirred until salts dissolved, The pH was adjusted to 6.0, The volume was filled to 5 L with distilled water, Storage in an airtight container.	FeSO_4_·7H_2_O (8 g L^−1^)—MnSO_4_·H_2_O (2.25 g L^−1^)—ZnSO_4_·7H_2_O (2 g L^−1^)—CuSO_4_·5H_2_O (1 g L^−1^)—(NH_4_)_6_Mo_7_O_24_·4H_2_O (0.23 g L^−1^)	Trace elements solution—pH: 6.0
ALCRI-Bio-Help	Prepare humic substances as above, Inoculated with *Azotobacter* sp. under controlled conditions for optimal growth, Maintained moisture and temperature to promote microbial activity, Stored in airtight containers at 4 °C until use.	Humic substances—*Azotobacter* sp.	Bio-fertilizer enriched with *Azotobacter*—nutrient-rich solution

## Data Availability

Data are available upon reasonable request from the corresponding author.
